# Supplementary material for: Patterns of Intron Gain and Loss in Fungi
Source: PLoS Biol. 2004 Nov 30;2(12):e422. doi: 10.1371/journal.pbio.0020422 (PMC532390; doi:10.1371/journal.pbio.0020422)
Supplement: Table S1 — Also available at http://genes.mit.edu/NielsenEtAl/. (4.3 MB ZIP). [file pbio.0020422.st001.zip › NielsenEtAl/html/623.html]

AN0134.1.NCU01613.1.MG03894.1.FG10756.1


```
 CLUSTAL W (1.82) Multiple Sequence Alignments - Introns Inserted


Sequence 1: NCU01613.1	718 aa
Sequence 2: MG03894.1	776 aa
Sequence 3: FG10756.1	786 aa
Sequence 4: AN0134.1	851 aa
Alignment Length: 889 aa
Number Identitical Residues: 230 aa
Alignment Score (without introns) 14021


MG03894.1 	------MSADYESFDAGRPGFVIGNHESGRWDALTDNHHSHVLASG0FNFVTAPVTNRHF
NCU01613.1	MSSNRGFNPESESFEQ-RPPFYIGQHDVQRHESPTDNQYGQILDSG0S----------HL
FG10756.1 	MASEDAGFDMSDGFGLQRPNFHIGYHNSNSNEPLTDIQYGHLLNHG0LAFVTTPITNTHF
AN0134.1  	--------MESE---SVAPSFCIGQHESKRTIPITSEVVQLAHESN0YDMLTTPITTPHF
          	           :      * * ** *:     . *.         .     ::. :. *:

MG03894.1 	RQRVVDLYNSHVKETQEFGLTD--AEKANPSLPGPIISTLTDDDTSLFPSSFVSAIIAYS
NCU01613.1	QERE--------QWVTERGLTD--AQKANPSLPLPTVPTLTDDDTSLFPSSYVGSLIAYA
FG10756.1 	RDRVFALVSEHLSTLSSNGEKP--TTTATGSRAEPILPPLTTKDTGLFPCAAVNTYTAVI
AN0134.1  	QSRVLSLLSSHLSNIQAVSHSDSGTLMTTENIRPLVMPQLGPADTHLTPNEAMSQLVGVT
          	:.*     ..  .     . . :.:  :. .     :. *   ** * *   :.   .  

MG03894.1 	SPWIDLCSEDPLVADLSRQALNLEVAYANFCGVRSIVIPGPRRDE----DAKGVAQYAR~
NCU01613.1	SPWIDLCSADPIISDISRQVLNLELAYANFCGSRTIIIPGPRQD-----DGRAVAQYAQ1
FG10756.1 	SPWIDLGSSNPIISSISRQVLNVEINYANFCGVRSIIIPGPRQDASIDGGNQSLAQYSR~
AN0134.1  	SSWIDLCSPDPLIADLSRQVFMLEVAYAAFCGIGYLLIPGPKLHHKG-MHSDGVMYYAR~
          	*.**** * :*:::.:***.: :*: ** ***   ::****: . .      .:  *:: 

MG03894.1 	AIQEAFLIATRLNFIIHMPMYREPNLEETDMLLSEAVGRQTKVAQKKE---IDLFSAWDT
NCU01613.1	TLSSLLAGDG----------SSSSNSKET---------VKTDAAKGTE---IDLFSTWDS
FG10756.1 	AVEEALTVGNRLTFLVQMPMYREPGQESKEVNISSLDVKTPTKTEGKE---IDLLAAWDS
AN0134.1  	AIQDALSLGPYIQFHIWLDMVDNQDLELDEMGDLAPLAREEFFDTEIEQPKIDLFGTWDA
          	::.. :                . . :  .                 *...***:.:**:

MG03894.1 	WHTIRSVNDYNVRLFVA1MRIPKRLPEKELQTRWFAEPLHYLTLGPNAFEKNRAGHPSLT
NCU01613.1	WHTIRTVCKYSGRLFVA~LRIPKRVPEKDLQERWFSEPLHYLTLDKKIFSLNKAGHPSLT
FG10756.1 	WHHVRSVCNYNTRLFVS1LEIPRVMPEKALQDRWFAEPLHYLTFSPSTFQANKAGFPSLS
AN0134.1  	WDAIRRTCKYHSRLFVA1LSLPKHLPPMAVQSRWHSEPVHLFTIDSNTFIKNQKGYPVLS
          	*. :* . .*  ****: : :*: :*   :* **.:**:* :*:. . *  *: *.* *:

MG03894.1 	KNHQEMINMYMRLKAYPWLIVYD~LG----PSKEELTRAEKAGSEFPTLAEAANG-----
NCU01613.1	RHHQDLINRYMRLKNHPWLILIN0------AVKENRKPYAPPYNEY--------------
FG10756.1 	KHHQNLIFSYMRLKNVPWILLCD~VGPDVSHLNESPQAPPTSQVEFPSLSEAETQNQLPK
AN0134.1  	KAHQALISRFMRLRTAPWILLCD~VG--PIPGVETDNASSLPGSEYPSLAQAAAS-----
          	: ** :*  :***:  **::: :  .       *       .  *:.: :.:        

MG03894.1 	--DSRIASNPEVAYLRHLERQQPPYGKLESPALISFQDWLQSPLQPLSDNLESSTYEMFE
NCU01613.1	-----------VDYMKYLERHQPPYSAMETPSLVSFQDWLQSPLQPLSDNLESATYEVFE
FG10756.1 	GQASQVKTNDYISYLRWLEDQQPPFTYLESPTLTSFQDWLQSPLQPLSDNLESATYEVFE
AN0134.1  	-IKKHHDPTPHLSYMRNLQSRQPPRTAIER-FGTGYQDYLQAPLQPLTVNLESITYEVFE
          	   .   ..  : *:: *: :***   :*     .:**:**:*****: **** ***:**

MG03894.1 	GDPVKYDQYEKAITEAMAEWKLLKKPTSGFNKETQE-PELVVTVAGAGRGPLVTRVLRAS
NCU01613.1	GDPVKYDQYEKAVAEAMLEWKKYNRPVSSVTPETPDGKDLIVAVAGAGRGPLVARVLRAA
FG10756.1 	GDPVKYSQYEIAVFEALTEWKELKKPLS-------KEGKVVVAVAGSGRGPLVTRALKAS
AN0134.1  	KDPIKYEWYERAIAKALSDWVEQKKPTSN------PDGRVVVAVVGAGRGPLVTRALKAS
          	 **:**. ** *: :*: :*   ::* *.          ::*:*.*:******:*.*:*:

MG03894.1 	AATGFPVQLWALEKNQNAYVYLLRQNERVWGGKVRVIKTDMREWDGIVPAGQKVTAENPA
NCU01613.1	ASTNTPIQLWALEKNQNAYVYLLRRNKREWNNSVTVIKTDMREWEGPRLAAN----PNII
FG10756.1 	EDAGVPIDMWAVEKNPNAYVYLLRQNELVWGGKVRVVKTDMRAWKGPVVSEDE--NGPVY
AN0134.1  	AQSGVEIDLWVVEKNPNAFVLLQRHNENLWGGKASLVHSDMRAWKGPRVRKSTTLSTEPV
          	  :.  :::*.:*** **:* * *:*:  *.... ::::*** *.*     .        

MG03894.1 	G---------------------------------SVDILVTELLGSFGDNELSPECLDGI
NCU01613.1	T---------------------------------KLDILVTELLGSFGDNELSPECLDGI
FG10756.1 	G---------------------------------KVDILISELLGSFGDNELSPECLDGI
AN0134.1  	GQSLGIEGQFLYTPDPNQKTADSPSLDAIEFEDSKIDIVVSELLGSFGDNELSPECLDGV
          	 .: . ...   :......::.:.: .: . ..:.:**:::******************:

MG03894.1 	QRHLAKPHGISIPHSYTAHLSPIAHPKLHAEISGRFATDPNSYNTPWVVRLFAIDFLASR
NCU01613.1	QRHIARPHGISIPYSYTAHLSPIAHPRIFADLSNRVVSDPNAFDIPWVTRLFQLAYVAQK
FG10756.1 	QHVMSTPHGISIPSSYTAHMSPISTPKIHGDILSRVPGDPHAFETPWVVRLFALDFVAER
AN0134.1  	NHLLNPVHGISIPASYTAHLTPISAPKLHADVTNQSITNPAAPETPYVVMLHAIDYLSTN
          	:: :   ****** *****::**: *::..:: .:   :* : : *:*. *. : ::: .

MG03894.1 	GVPGKPRFQQAWEFVHPVRLPFAEKWAAEHGPHRRPIRGGGGAMNLSSGLN-EHNARHCH
NCU01613.1	-VPKHGRFQQAWEFVHPVEVSRADDFSAQNGRARKYVTHGSGSMYGSSGIN-EHNARHCH
FG10756.1 	-VPNKPRFQEAWEFSHPIPESSLAALEAK--RSGGVVGGGGGSMAGAAGAN-DHNSRYCH
AN0134.1  	-QSDASAGNPARSSVATVPYEPTTPFVQTAWSFSHPNRDIPPQPASTSMISNAHNVRRTR
          	  .     : * .   .:                            ::  .. ** *  :

MG03894.1 	LTFVCGRRGVMHGLAGYFESTLYASQVQGKEKELVEISILPEQIDRKSKDMISWFPIYFP
NCU01613.1	LTFVCPTRGVIHGLAGYFESVLYKHQEEGKTP--VEISILPDQIDRKSKDMISWFPIFFP
FG10756.1 	LTFVCRTRGVTHGLAGYFESTLYESQIPDNKGAKIEISTHPERIDDKSKDMISWFPIFFP
AN0134.1  	LTFPVPNRGVCHGLAGYFETVLYRD---------VELSTNPVTMDSKSANMISWFPIYFP
          	***    *** ********:.**           :*:*  *  :* ** :*******:**

MG03894.1 	LN0KPLYFPEDAELEVSMWRQTDDTRVWYEWMVEAYLWVSQT------------------
NCU01613.1	LR0QPLYFPQDTELEVSMWRQTDDTRVWYEWMVEVYAWVGPK------------------
FG10756.1 	LK0KPLYFPADTELEVSMWRQTDDTKVWYEWLVEAYTWVGAS------------------
AN0134.1  	LK0TPLNVPDNGEIVATMYRQTDDRKVWYEWMVEVFALEGGSEPASASAPASERIAPVMS
          	*.  ** .* : *: .:*:***** :*****:**.:   . ...:::::.::.  :.  :

MG03894.1 	---------------------------------QRVKIGASDLHSSRKIACLM-
NCU01613.1	---------------------------------TRIKVGASEMHSSRKVACIFG
FG10756.1 	---------------------------------TRVKVASSDLCSSRQVACLM-
AN0134.1  	GARTISASADSAHNKDITADSYSRLAQKKARGPRRVRVGMSDLHSSIKDGCLM-
          	.: : ::::.:: ... ::.: :  :...: .. *:::. *:: ** : .*::
```
